# Where do I go? Decoding temporal neural dynamics of scene processing and visuospatial memory interactions using convolutional neural networks

**DOI:** 10.1167/jov.25.10.15

**Published:** 2025-08-28

**Authors:** Clément Naveilhan, Raphaël Zory, Stephen Ramanoël

**Affiliations:** 1 Université Côte d'Azur, LAMHESS, Nice, France; 2 Institut Universitaire de France (IUF), Paris, France; 3 Sorbonne Université, INSERM, CNRS, Institut de la Vision, Paris, France

**Keywords:** visual perception, scene, spatial memory, temporal dynamic, EEG, grad-CAM

## Abstract

Visual scene perception enables rapid interpretation of the surrounding environment by integrating multiple visual features related to task demands and context, which is essential for goal-directed behavior. In the present work, we investigated the temporal neural dynamics underlying the interaction between the processing of bottom-up visual features and top-down contextual knowledge during scene perception. We asked whether newly acquired spatial knowledge would immediately modulate the early neural responses involved in the extraction of navigational affordances available (i.e., the number of open doors). For this purpose, we analyzed electroencephalographic data from 30 participants performing interleaved blocks of a scene memory task and a visuospatial memory task in which we manipulated the number of navigational affordances available. We used convolutional neural networks coupled with gradient-weighted class activation mapping to assess the main electroencephalographic channels and time points contributing to the classification performances. The results indicated an early temporal window of integration in occipitoparietal activity (50–250 ms post stimulus) for several aspects of visual perception, including scene color and number of affordances, as well as for spatial memory content. Moreover, a convolutional neural network trained to detect affordances in the scene memory task failed to generalize to detect the same affordances after participants learned spatial information about goal position within the scene. Taken together, these results reveal an early common window of integration for scene and visuospatial memory information, with a specific and immediate top-down influence of newly acquired spatial knowledge on early neural correlates of scene perception.

## Introduction

Visual scene perception is a critical cognitive function that enables individuals to rapidly encode, interpret, and interact with their environment. This process requires the integration of low-, mid-, and high-level visual information (e.g., object features, spatial configurations, and landmarks) (Bartnik & Groen, 2023) to support efficient and adaptive behavior in dynamic settings such as natural environments (Epstein & Baker, 2019). The complexity of visual perception becomes even more pronounced in everyday life when we consider the variety of goals, tasks, contexts, or prior knowledge that modulate scene processing and its neural correlates (Bar, 2009; Bar et al., 2006; Kay, Bonnen, Denison, Arcaro, & Barack, 2023; Malcolm, Groen, & Baker, 2016; Nau, Schmid, Kaplan, Baker, & Kravitz, 2024; Ritchie, Wardle, Vaziri-Pashkam, Kravitz, & Baker, 2024). Therefore, a deeper understanding of the neural dynamics underlying scene perception, especially the interaction between bottom-up (e.g., visual features) and top-down (e.g., prior knowledge) information, is essential for elucidating the mechanisms of visual cognition.

In this context, the processing of navigational affordances available in visual scenes represents a promising theoretical and methodological framework (Bartnik, Vukšić, Bommer, & Groen, 2024; Djebbara, Fich, Petrini, & Gramann, 2019; Gregorians & Spiers, 2022; Naveilhan, Saulay-Carret, Zory, & Ramanoël, 2024). Initially proposed to be automatically extracted solely by bottom-up mechanisms during scene perception (Bonner & Epstein, 2017; Harel, Nador, Bonner, & Epstein, 2022), recent findings also suggest the influence of top-down processes such as contextual information on scene processing (Aminoff & Tarr, 2021; Choi, McCloskey, & Park, 2020; Naveilhan et al., 2024). For example, Naveilhan et al. (2024) reported that learning the position of a goal situated in an adjacent room interfered with the number of navigational affordances available (i.e., open doors in a room). Precisely, participants exhibited a linear decrease in task accuracy as the number of doors increased, a pattern that was not present before participants learned this spatial information. However, the neural mechanisms underlying this effect remain poorly understood. Indeed, whereas Naveilhan et al. (2024) proposed that learning contextual information might modulate early neural markers of visual scene processing, their results did not clarify how scene perception and visuospatial memory interact at the neural level to produce the behavioral effects observed. A possible explanation resides in the fact that these analyses were restricted to only few occipitoparietal electrodes reported to be involved in the extraction of affordances (Bonner & Epstein, 2017; Harel, Groen, Kravitz, Deouell, & Baker, 2016; Harel et al., 2022; Kaiser, Häberle, & Cichy, 2020; Kamps, Chen, Kanwisher, & Saxe, 2024). Despite the interest of these restricted analyses, this may also have constrained the detection of broader, integrative brain dynamics that likely underpin complex visuospatial behaviors (Groen, Silson, & Baker, 2017; Kravitz, Saleem, Baker, & Mishkin, 2011). Thus, the neural correlates of this interaction between scene and visuospatial memory information remain elusive.

Recent advances in signal processing and supervised learning with neural networks now make it possible to consider multiple brain regions that support complex dynamics for visual perception. These computational approaches enable the extraction of spatiotemporally distributed neural patterns beyond what is accessible with traditional event-related potential (ERP) averaging. They offer a data-driven means to disentangle the contributions of distinct brain regions over time without prior assumptions about electrode relevance, but also how the information supporting classification generalize to different contexts. Notably, Orama and Motoyoshi (2023) trained a convolutional neural network (EEGNet Lawhern et al., 2018) to classify both scene categories and their global properties (e.g., naturalness, openness, roughness) based on electroencephalographic (EEG) data. By applying gradient-weighted class activation mapping (Grad-CAM; Selvaraju et al., 2017), the authors reported that early EEG signals from occipital electrodes were crucial for classifying openness, whereas later signals from frontal electrodes were crucial for determining naturalness and scene categories. Dwivedi, Sadiya, Balode, Roig, and Cichy (2024) further explored this complex sequencing of scene perception using representational similarity analysis to compare neural responses across occipital electrodes with computational models of two-dimensional, three-dimensional, semantic features, and navigational affordances. Their results showed that visual features are processed earlier in the temporal sequence (130–170 ms post stimulus) than navigational affordances (approximately 300 ms), suggesting hierarchical processing in line with the findings of the Orima group. However, these results also seem to be partially inconsistent with those of Harel et al. (2022), who showed that navigational affordances were processed earlier (approximately 230 ms). A possible explanation is based on the fact that in previous protocols participants were passively presented with the scene (Bonner & Epstein, 2017; Harel et al., 2022), whereas in Dwivedi et al. (2024) participants had explicitly engaged in navigational affordance processing.

These differences in task engagement, contextual information, and the features of the visual scenes used could influence the temporal dynamics of navigational affordance extraction, as previously reported only at the behavioral level (Naveilhan et al., 2024). Extending this notion of top-down influence to the neural level, Enge, Süß, and Rahman (2023) proposed that acquiring semantic knowledge about an object's function can immediately alter its visual processing. Using EEG, they demonstrated that, when participants viewed unfamiliar objects paired with a functional keyword rather than a filler, semantic insight immediately enhanced the high-level N170 component (150–200 ms), reduced the N400 (400–700 ms), and, during subsequent uncued viewing, increased the early P1 response (100–150 ms). These findings indicate that semantic knowledge can rapidly modulate both low- and high-level perceptual representations during goal-directed behavior, aligning with models of visual perception (Bar, 2009; Bar et al., 2006) proposing that frontal context-driven predictions preactivate spatial templates in high-level visual areas. However, this mechanism, embedding goal position information into the initial feedforward sweep, was entirely overlooked by the univariate ERP analysis in Naveilhan et al. (2024), which focused exclusively on P2 amplitude at occipitoparietal electrodes. Thus, within the domain of scene perception, the temporal dynamics of the neural correlates underlying the interaction between scene information processing and prior spatial information remain elusive and represent a major challenge for current research in visual cognition (Nau et al., 2024; Ritchie et al., 2024).

To address this issue, we conducted novel analyses using convolutional neural networks (CNNs) applied to the previously acquired dataset described above (Naveilhan et al., 2024). Participants completed scene perception and visuospatial memory tasks during which they first viewed the scenes, then learned the position of a goal and were subsequently asked to retrieve it. Throughout the experiment, we systematically manipulated the number of available navigational affordances while controlling for low-level visual features across conditions. We included all 64 available electrodes to investigate the global dynamics of the expected interaction between task-relevant top-down (i.e., prior spatial knowledge) and bottom-up information processing (i.e., visual features such as the number of opened doors). The aim of the present study was to provide new insights into the temporal neural dynamics underlying task-specific visuospatial representations by leveraging the complexity of CNNs to disentangle the rich spatiotemporal structure of EEG data. Specifically, we hypothesize that top-down information will modulate the early neural signature of scene perception, including neural correlates of visual scene features, extending results from Enge et al. (2023) to scene processing. In this sense, we expect that processing of prior spatial knowledge will modulate early occipitoparietal activity (approximately 200 ms), allowing the CNN to classify whether contextual information has been acquired. We also propose that this time window carries specific representations of the previously learned goal position, suggesting an early common integration for both scene and visuospatial memory information. Finally, we hypothesize that learned spatial information will modulate the early neural signature of affordance processing, even when visual features remain identical. Specifically, we expect that a CNN trained to classify navigational affordances without prior spatial knowledge will fail to generalize to conditions after participants have learned goal-related information, even though the visual scene features are the same across conditions. This would suggest that visuospatial memory information interacts with the processing of visual scene features, thereby modifying how affordances are represented at the neural level.

## Methods

### Participants

In the present study, we conducted a reanalysis of a dataset from a previously published study (Naveilhan et al., 2024). EEG data were collected from 30 young adults (mean age, 24.31 ± 0.65 years; range, 19–31 years; 16 females). Sample size was determined a priori using G*Power (version 3.1.9.7; Faul, Erdfelder, Lang, & Buchner, 2007) based on a previous EEG study of scene perception (Naveilhan et al., 2025), which indicated that a minimum of 23 participants was required to achieve statistical power of 0.95 at an alpha level of 0.05. The study was approved by the local ethics committee (CERNI-UCA opinion no. 2021-050), and all participants gave informed consent prior to participation. They were all right handed, had normal or corrected-to-normal vision, and had no history of neurological or cognitive disorders.

### Stimuli and procedure

**Figure 1.**
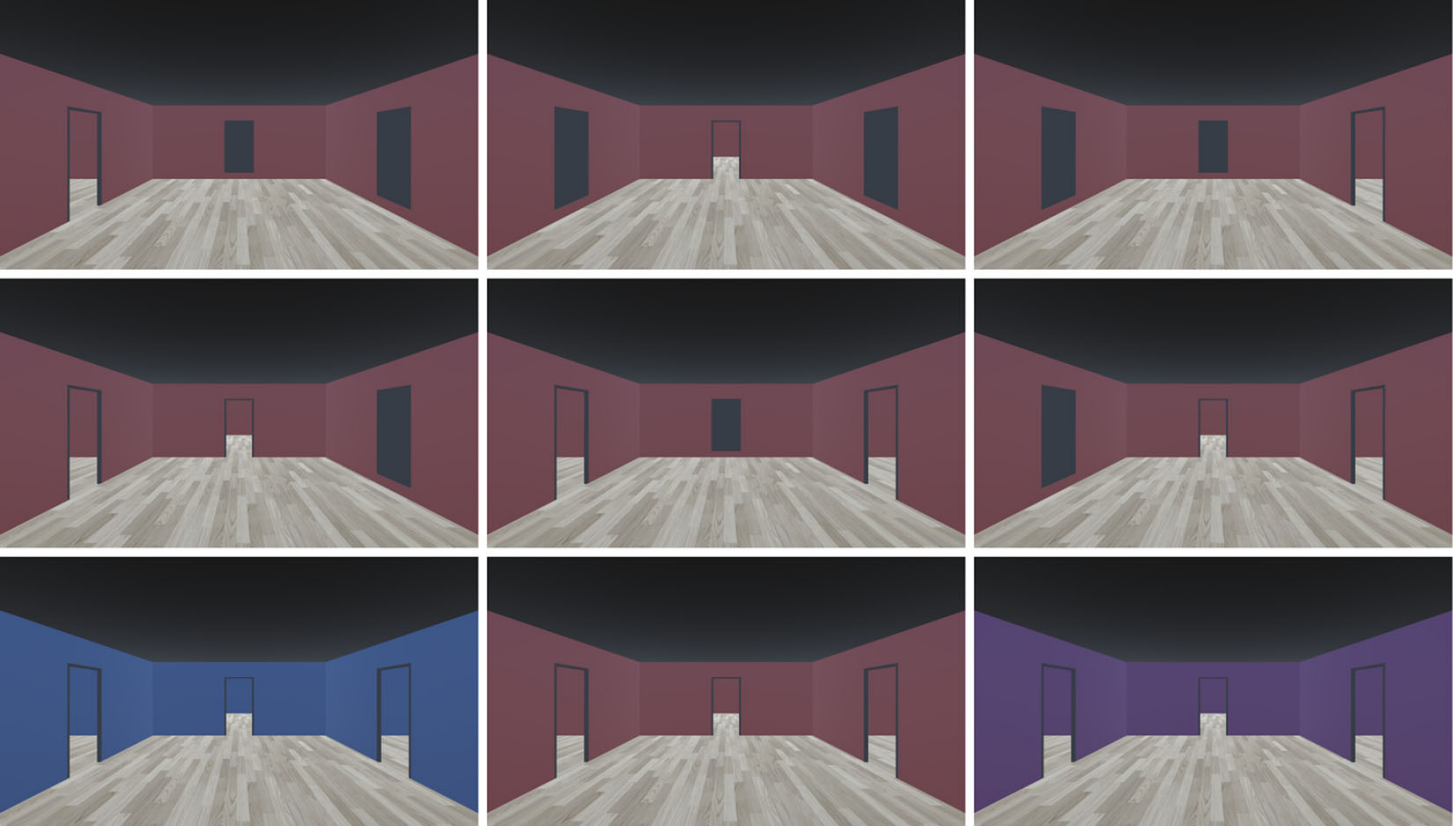
Presentation of a subset of the stimuli used, showing the seven possible affordance configurations and three of the eight wall colors included in the protocol. The complete set of stimuli is available in the OSF repository.

The experiment used visual stimuli developed with Unity Engine (v2019.2.0.0f1) and presented on an iiyama ProLite B2791HSU monitor (1,920 × 1,080 resolution, 30–83 Hz) positioned 60 cm from the participants. Stimulus presentation was controlled by PsychoPy (v2022.13) running on a Dell Precision 7560 workstation with an Intel Xeon W-11955 processor. The stimuli consisted of images of simple rectangular rooms, each containing either a door (i.e., a navigational affordance) or a gray rectangle on three visible walls. These designs were adapted from previous studies (Bonner & Epstein, 2017; Harel et al., 2022). To avoid potential associations between door locations and wall colors, seven different door configurations and seven wall color variations were used, resulting in 49 unique stimuli. All stimuli are publicly available in the OSF repository, and a subset is presented in Figure 1.

Participants completed two tasks: a scene memory task and a spatial memory task. In the protocol they completed six similarly structured blocks, and within each block, stimulus presentation was pseudorandomized to maintain engagement. Each block began with the scene memory task, a one-back paradigm in which participants indicated whether the current scene matched the previous one based on wall color alone and responded with a designated key if a match was detected. This task consisted of 64 images depicting four door configurations in two environments (i.e., two wall colors), with each image presented eight times. Participants then performed the spatial memory task. First, they passively viewed a guided navigation sequence through a series of rooms to learn the location of a hidden target that could appear on the left, center, or right. Importantly, each wall color was consistently associated with a specific target direction. Participants were then presented with the same pictures as those used in the scene memory task and asked to indicate the remembered goal direction as quickly and accurately as possible using a USB keyboard (e.g., in the red room the goal is on the right and on the left in the blue one). Although the visual stimuli were the same in both tasks, the spatial memory task required participants to integrate spatial contextual information gained during the navigation phase. In both tasks, accurate performance depended on attention to the color of the wall, thus aligning visual input and attentional demands across tasks. The spatial memory task always followed the scene memory task within each block to prevent contamination of the scene memory task by prior spatial learning.

For each task, images were displayed for 1 second, followed by a fixation cross lasting between 0.5 and 1.0 second, and auditory feedback for responses. Over the course of the experiment, participants viewed a total of 1,540 images (770 per task), during a unique session lasting approximately 55 minutes. Each task involved 7 possible affordances, resulting in 110 repetitions of each configuration per participant, with variations in wall color. With data from 30 participants, this resulted in an average of approximately 3,300 trials per class (e.g., left door opened in the scene memory task). This number is comparable with the 3,337 trials per class reported in previous work (Orima & Motoyoshi, 2023) and the order of magnitude suggested for such analyses in deep learning-based EEG studies (Roy et al., 2019).

### EEG preprocessing

EEG data were acquired at a sampling rate of 500 Hz using a 64-channel waveguard cap with active wet electrodes, connected to an eego mylab amplifier (ANT Neuro) and digitized at 24-bit resolution. The reference electrode was positioned at CPz, and the ground electrode at AFz. Electrode impedances were maintained below 10 kΩ, with the majority under 5 kΩ. EEG recordings were synchronized with stimulus presentations using LabRecorder in the Lab Streaming Layer (Kothe et al., 2024).

Because preprocessing steps have been proposed to play an important role in the decoding capabilities of EEGNet (Kessler, Enge, & Skeide, 2024), we opted for an open source and fully reproducible pipeline implemented in MATLAB (R2024a), namely, the BeMobil pipeline (Klug et al., 2022) using EEGLAB v2024.2 (Delorme & Makeig, 2004). Non-experimental segments and highly artifacted portions of the data were manually excluded and the signals were downsampled to 250 Hz. Line noise was automatically detected and removed using Zapline Plus (Klug & Kloosterman, 2022). Bad electrodes were detected based on a correlation threshold with adjacent electrodes of 0.8 and a maximum downtime of 0.6, and then interpolated using spherical interpolation methods, with an average of 3.10 ± 2.58 electrodes removed per subject. The data were referenced to the common average. Additional artifact cleaning was performed in the time domain using artifact subspace reconstruction with a cutoff threshold of 20 (Chang, Hsu, Pion-Tonachini, & Jung, 2020), resulting in the removal of an average of 7.51 ± 3.52% of the data points. We included artifact subspace reconstruction in the BeMoBIL pipeline to remove high-amplitude, non-stereotypical noise bursts early on (Delaux et al., 2021) stabilizing the subsequent independent component analysis decomposition, which then isolates structured artifacts (Chang et al., 2020).

Subsequently, the data were temporally high-pass filtered at 1.5 Hz and adaptive mixture independent component analysis decomposition was applied with 10 rejection iterations and a sigma threshold of 3 (Klug, Berg, & Gramann, 2024; Klug & Gramann, 2021). Dipole fitting was performed using DipFit, and independent components were classified using ICLabel with default parameters (Pion-Tonachini, Kreutz-Delgado, & Makeig, 2019). Components identified as muscle, line noise, eye, or heart artifacts were excluded, and only those classified as brain or other with residual variance less than 15% were retained (Delorme, Palmer, Onton, Oostenveld, & Makeig, 2012). All the computed adaptive mixture independent component analysis information and dipole fitting were then copied to the initial preprocessed unfiltered dataset. The cleaned data were then band-pass filtered between 0.3 and 50.0 Hz, and epochs from 1 second before to 2 seconds after stimulus onset were extracted. Epochs containing artifacts larger than 100 µV were excluded, resulting in an average of 1,461 ± 90.02 epochs per subject, balanced across conditions. This final check ensured high signal quality which was proposed to help improve model robustness (e.g., see Kessler et al., 2024; Roy et al., 2019)

### EEGNet and Grad-CAM procedure

#### Data preparation

Data were exported from MATLAB to Python for subsequent analyses using an EEG-based classification framework implemented with PyTorch (Paszke et al., 2019). Analyses were performed on an NVIDIA RTX 4090 GPU with 24 GB of VRAM using CUDA 12.6. Each dataset was normalized at the subject level and balanced to ensure equal class representation (e.g., we ensured an equal number of spatial and scene memory trials for the first model). We used full EEG epochs, from image onset to 1 second post stimulus (250 time points), with baseline correction applied between −200 ms and 0 ms, following standard ERP preprocessing practices and recommendation for neural network (Kessler et al., 2024). Both electrodes of the right and left mastoid were removed from the dataset; these electrodes were highly artefacted for most of the participants.

#### EEGNet procedure

EEG decoding was performed using the EEGNet convolutional architecture (Lawhern et al., 2018), which efficiently combines depthwise spatial and separable temporal convolutions. Each trial (61 electrodes × 251 time points) was input to a first temporal convolution layer that extracted frequency-specific features, followed by a depthwise–spatial convolution and a pointwise separable convolution. This sequence consisted of *ConvBlock 1*, whose output was then batch-normalized (Ioffe & Szegedy, 2015), activated with an exponential linear unit (Clevert, Unterthiner, & Hochreiter, 2016), averaged, and regularized by two-dimensional dropout (Srivastava, Hinton, Krizhevsky, Sutskever, & Salakhutdinov, 2014). A second, identical *ConvBlock 2* further refined spatiotemporal patterns. The resulting feature maps were flattened and passed to a fully connected layer, generating one logit per class (e.g., two tasks or seven affordance categories). By alternating temporal and spatial convolutions, batch normalization, exponential linear unit activations, average pooling, and dropout, EEGNet captures both when (temporal patterns) and where (spatial patterns across electrodes) relevant EEG features occur, while keeping the parameter count low for rapid, single‐trial classification (Lawhern et al., 2018). Batch normalization accelerates convergence and stabilizes training (Ioffe & Szegedy, 2015), exponential linear unit mitigates vanishing gradients (Clevert et al., 2016), and channel-wise dropout prevents overfitting (Srivastava et al., 2014). To compensate for class imbalances and promote generalization, we optimized the network using class-weighted cross-entropy loss (Cui, Jia, Lin, Song, & Belongie, 2019).

#### Evaluation of the trained model

After training the model on 80% of the full pooled dataset (i.e., containing data from all the subjects) with early stopping, performance was evaluated on the 20% remaining dataset using five-fold cross-validation for each individual subject. Classification accuracy was then compared with chance levels using a binomial cumulative distribution approach. For each analysis, a binomial distribution was computed using MATLAB's *binoinv* function, taking into account the predefined significance level (α = 0.05), the number of predictions (the average number of classifications made per participant), and the number of classes (2 for task predictions, 7 for affordances, and 8 for wall color). The binomial method was used to determine statistical significance, as it is comparable in reliability to permutation testing for datasets with more than 100 trials, but does not have the extensive computational requirements of permutation testing (Combrisson & Jerbi, 2015; Vahid, Mückschel, Stober, Stock, & Beste, 2020). We then used 100,000 iterations of bootstrap resampling to calculate the mean difference between model accuracy and a random sample drawn from the generated binomial distribution. From the bootstrap distribution of the mean differences between accuracy of the model and chance, we derived a 95% confidence interval and a *p* value, which are presented in the Results.

#### GradCAM procedure

Finally, Grad-CAM (Selvaraju et al., 2017) was used to visualize the contributions to classification by focusing on activations from the second convolutional block. This approach was informed by previous work demonstrating that applying Grad-CAM to intermediate convolutional layers in EEGNet effectively highlights spatiotemporal patterns relevant to cognitive tasks, such as scene perception (Orima & Motoyoshi, 2023). By targeting the second convolutional block, the visualization captures mid-level features that balance spatial and temporal information, providing more interpretable insights into the neural dynamics underlying scene processing. Specifically, gradients of predicted values were computed with respect to feature map activations, resulting in two-dimensional localization maps (electrodes and time points). These maps were processed through rectified linear unit layers, normalized, and averaged across participants to identify the key EEG channels and time points that most significantly contributed to the classifications, as illustrated in the Results with the relative importance score (a value normalized between 0 and 1). To ensure the robustness and generalizability of our findings, all analyses were repeated five times, and the median results were reported. Detailed information about the different models architectures and example code are available in the study's OSF repository.

#### Hyperparameter selection

In previous work using a similar architecture (Orima & Motoyoshi, 2023), the primary limitation identified was the arbitrary selection of hyperparameters. To overcome this issue, the present study adopts a more systematic approach, with hyperparameter selection using Bayesian optimization and HyperBand (Falkner, Klein, & Hutter, 2018). Using Optuna's Tree‐structured Parzen Estimator sampler and Hyperband pruner (Akiba, Sano, Yanase, Ohta, & Koyama, 2019; Li, Jamieson, DeSalvo, Rostamizadeh, & Talwalkar, 2018), we explored a search space encompassing training epochs, batch sizes, learning, weight decays, dropout rates, temporal filters in ConvBlock 1, spatial filters in ConvBlocks 2 and 3, and early stopping patience. To ensure robustness, this entire search–train–test procedure was repeated five times, yielding consistent accuracy across all repetitions.

## Results

### Early EEG signal is modulated by visuospatial memory information

**Figure 2.**
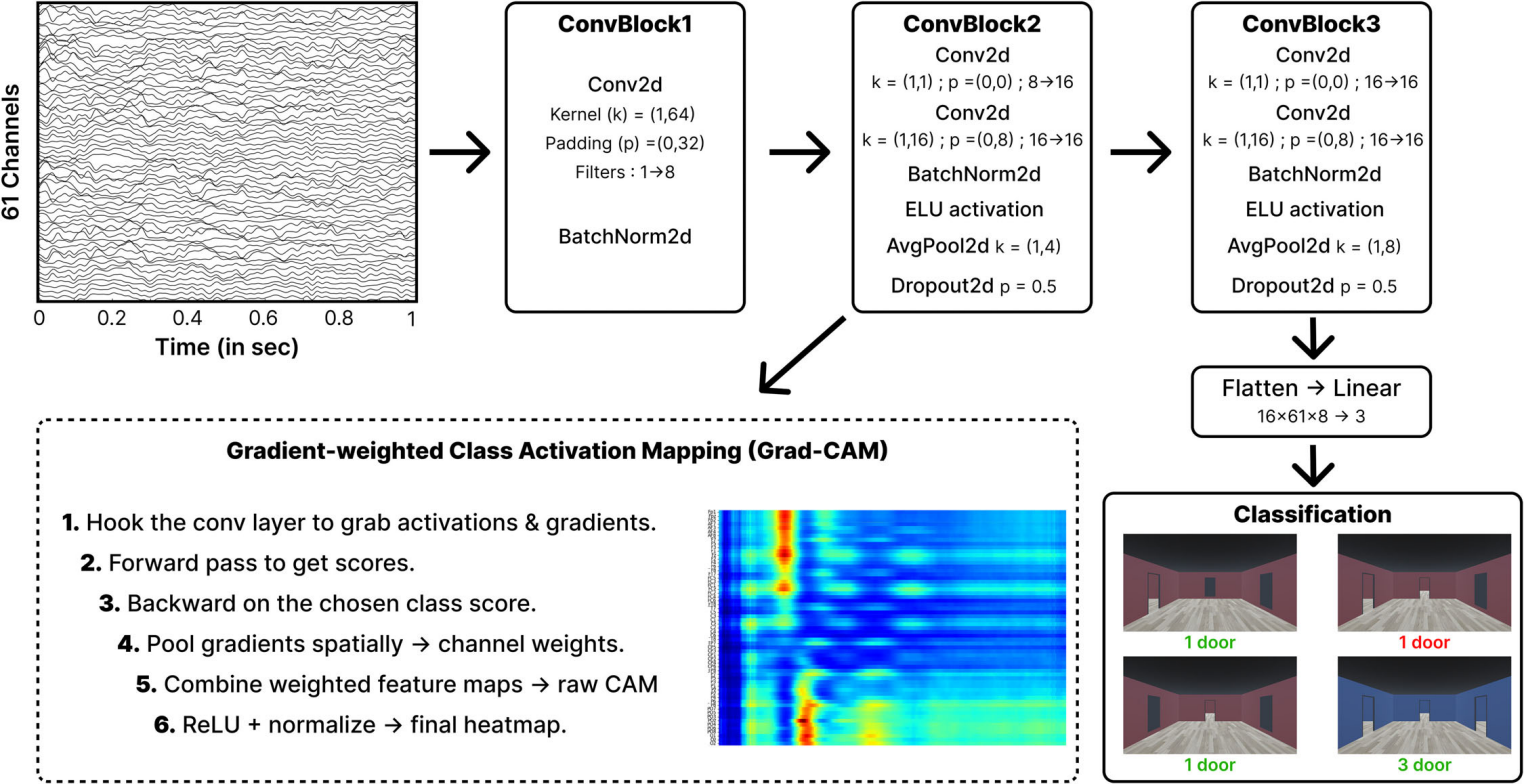
Schematic representation of the CNN architecture and interpretability pipeline. The input consists of a 61-channel EEG epoch with a duration of 1 second. It is then processed through three convolutional blocks and a fully connected layer to predict one of three door-count categories. For example here, the one door in red has been poorly classified, which would lead in this example of a 75% accuracy of the model. To enhance interpretability Grad-CAM heatmaps were used to visualize the contributions to classification by focusing on activations from the second convolutional block.

In the first analysis, we aimed to test whether learning contextual information (i.e., knowing that one of the opened doors hides a goal) modulates early neural markers of scene processing in a top-down manner. We also sought to determine whether this modulation is restricted to occipitoparietal regions, as suggested by Naveilhan et al. (2024), or whether it engages a broader network, including frontal regions. To investigate this, we trained a CNN on balanced data to classify the task participants were performing: either the scene memory task (before learning the goal's position) or the spatial memory task (after learning it). For each participant, we performed five-fold cross-validation and applied Grad-CAM to the second layer of the trained CNN (Figure 2) to identify the time points and electrodes most relevant for task classification. Here, we argue that, if the CNN can reliably distinguish whether participants have previously learned the goal's position, this would suggest that early neural markers of scene processing are indeed modulated by learning spatial contextual information, given that the visual stimuli remained identical across both conditions.

**Figure 3.**
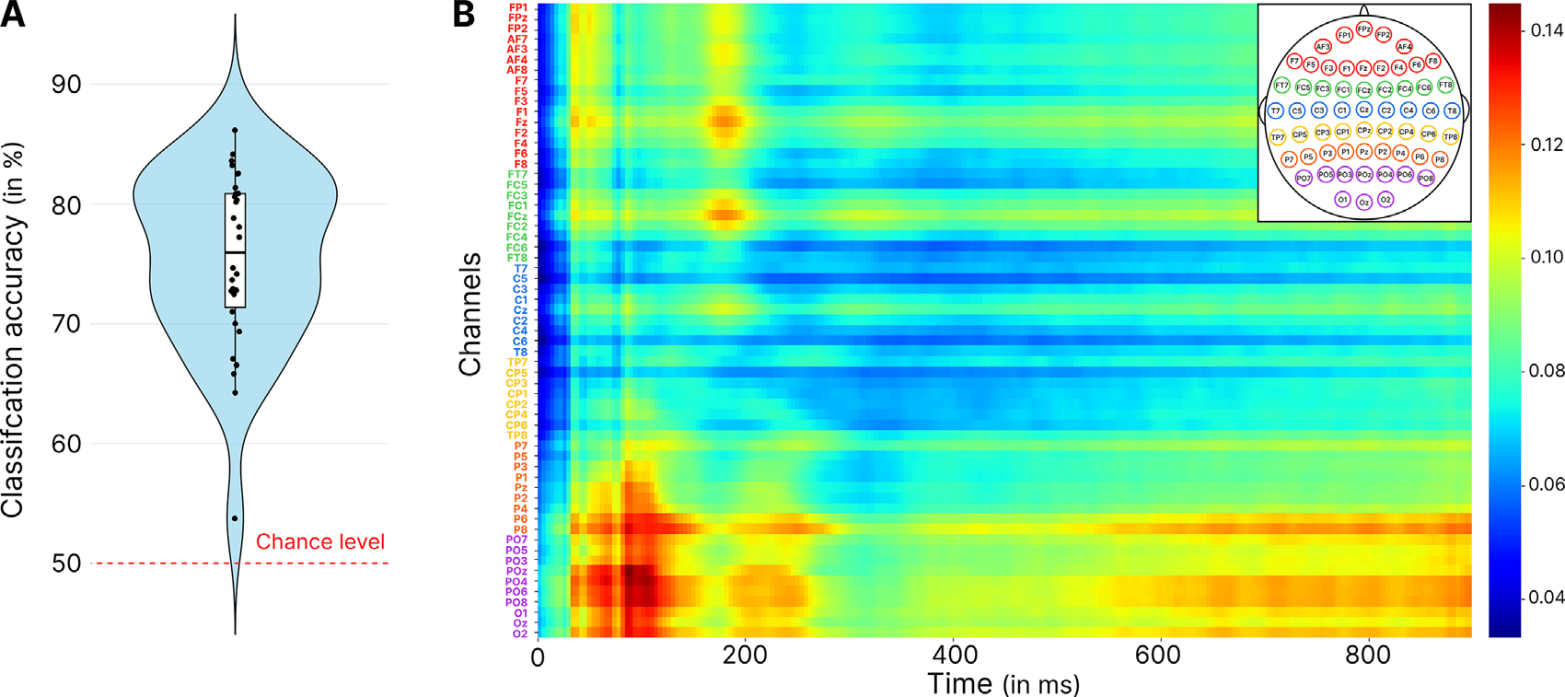
Results of the EEGNet classification and Grad-CAM analysis, for the CNN trained on the entire merged dataset to classify the task being performed. (**A**) Violin plot showing the accuracy of the model. (**B**) Grad-CAM results showcasing the time-channel points in the second CNN layer that most significantly contributed to the classification. The colorbar represents the relative importance score, normalized between 0 and 1. The most contributive points are in red, and the colorbar represents arbitrary units. The channels are arranged sequentially from frontal to occipital regions.

Looking at the accuracy of the model (Figure 3A), we find an average classification accuracy of 75.39 ± 7.28%. Bootstrapping analysis using the binomial distribution showed that the accuracy of the model was better than the 50% chance level (95% confidence interval [CI] of the mean difference from chance: 95% CI, 19.40–31.45; *p* < 0.0001). This result suggests that learning contextual information modified the neural correlates of scene perception allowing the network to classify the task. In order to identify which time points and EEG channels contributed most, we then looked at the Grad-CAM results (Figure 3B). These results highlight that the activity of a cluster of occipitoparietal electrodes (P8, POz, O1, O2, P6, PO4, PO6, PO7, PO8, and Oz) between 50 and 180 ms after stimulus presentation contributed the most to task classification, a result that may reflect differences in visual stimuli processing. The same electrodes also seemed to be involved later between 200 and 250 ms. Finally, later activity appeared after 600 ms, which also enabled the classification of the task, reflecting the onset of motor activity to produce the response.

In a second step, we extracted the EEG activity of participants involved in the spatial memory task (i.e., when they had to retrieve the target). We selected only trials in which participants were presented with two open doors and in which they successfully retrieved the position of the goal (93.47 ± 0.58% of trials per participant). This ensured that the network had sufficient trials and used only the EEG activity associated with goal direction information as support for the classification.

**Figure 4.**
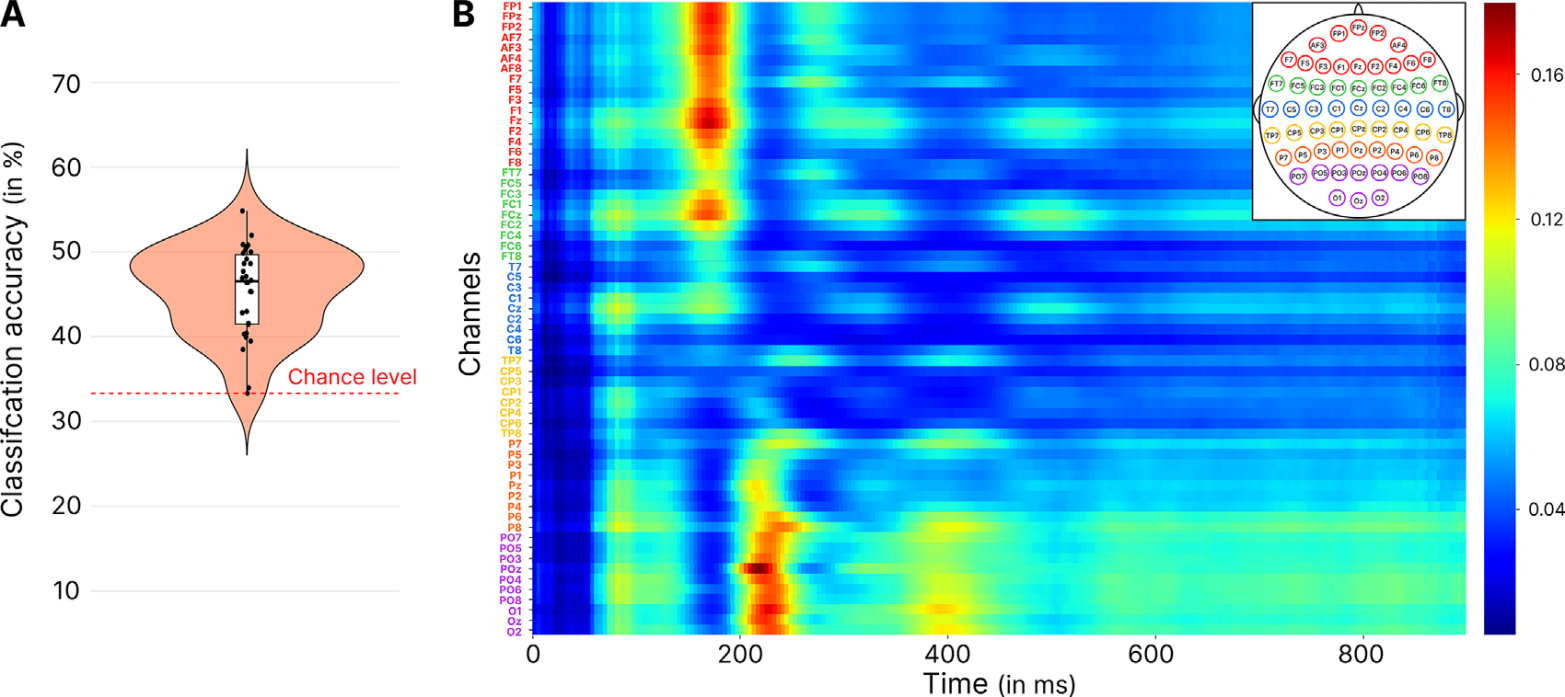
Outcomes of the EEGNet classification and Grad-CAM analysis, for the CNN trained on the dataset with the two affordances conditions to classify the goal position. (**A**) Violin plot displaying the model's accuracy. (**B**) Grad-CAM results indicating the time-channel points in the second CNN layer, with those making the highest contribution to the classification presented in red.

The accuracy of the model (Figure 4A) indicated that on average the network classified 45.41 ± 5.37% of the tested trials correctly, performing better than the 33% expected by chance (95% CI, 3.74–13.63; *p* = 0.0004). The Grad-CAM results (Figure 4B) highlighted activity in frontal electrodes between 180 and 200 ms, as well as activity in occipitoparietal electrodes between 200 and 220 ms as the most contributive to the goal position identification. These results suggest that early EEG activity also contains information regarding the position of the goal, irrespective of the visual features of the scene. This interpretation was further strengthened when we performed a similar analysis to detect the position of the goal, but in the three-affordance condition. Here, we also found that the model accurately classified the position of the goal (mean classification accuracy = 48.67 ± 6.54%; 95% CI, 6.95–16.89; *p* < 0.0001), despite the fact that there was no visual information in the presented scene allowing to distinguish between conditions. Together, these findings indicate that early EEG activity also encodes information about the goal's position, regardless of the visual characteristics of the scene.

### Prior spatial knowledge modulates early neural activity related to affordance processing

In this second analysis, we delve further into the previous results suggesting a modulation of early neural markers of scene processing following the learning of contextual information. First, we used EEG activity from participants performing the scene memory task and trained a model to detect affordances within the scene (i.e., the number and position of the open doors). Based on previous findings (Harel et al., 2022), we hypothesized that the model would perform above the chance level, confirming that navigational affordances are extracted automatically from visual scenes (Bonner & Epstein, 2017). Once the model was trained, we then used it to test our main hypothesis: that the neural correlates of affordance extraction are modified after participants learn that one of the doors leads to a goal. To test this, we applied this model to EEG data from the spatial memory task, where participants had learned the location of the goal. We argue that, if the model fails to generalize to this task, it would suggest that the information supporting decoding was modulated by learned spatial contextual information, providing further evidence for top-down modulation.

**Figure 5.**
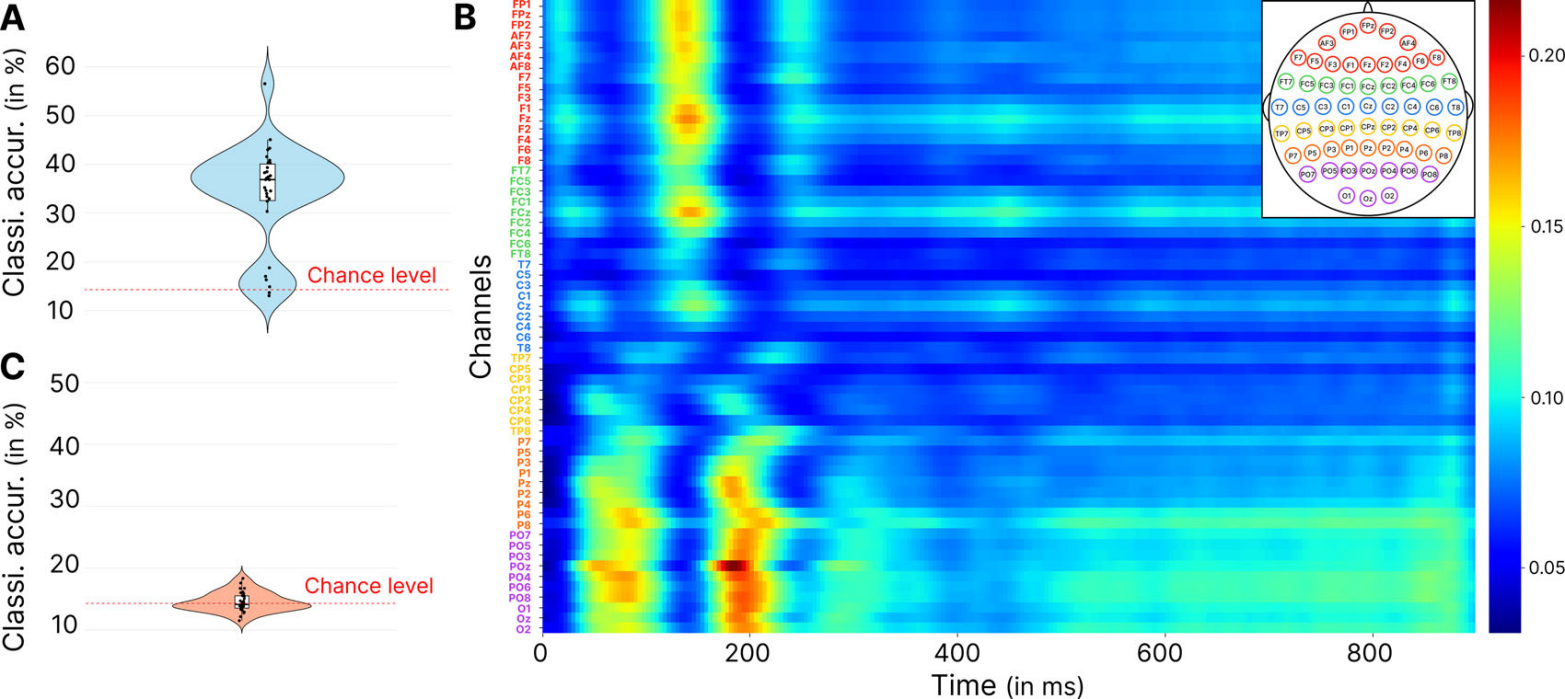
Results of the EEGNet classification and Grad-CAM analysis, for the CNN trained on the data of participants performing the scene memory task to detect the position of the affordances. (**A**) Violin plot showing the accuracy of the model during the scene memory task. (**B**) Grad-CAM results highlighting the time-channel points in the second CNN layer that contribute most to the classification. The most contributive points are in red (**C**). Violin plot of the accuracy for the model tested on the data of participants performing the spatial memory task.

The model tested on the scene memory task (Figure 5A) showed an accuracy of 35.80 ± 10.45%, and bootstrap testing indicated that it performed better than the 14.29% chance level (95% CI, 10.88–23.68; *p* < 0.0001). However, when we tested the model on EEG data from the spatial memory task to test the generalization of the model trained on the scene memory (Figure 5C), the classification accuracy dropped to 13.59 ± 1.78% and was no longer higher than chance level (95% CI, −7.41 to 3.11; *p* = 0.77). These results suggest that the information from neural activity enabling the classification of the position of affordances is modulated by spatial learning. The results of the Grad-CAM analysis (Figure 5B) indicated that the most contributive time points for the classification were once again located in the occipitoparietal region at approximately 200 ms, as previously suggested by modulation of the P2 component in ERP analyses (Harel et al., 2022). Interestingly, earlier occipitoparietal activity (50–100 ms) and frontal activity (150–180 ms) were also involved to a lesser extent in the classification, replicating previous findings on the neural correlates of low-visual feature processing such as the openness of visual scenes (Greene & Oliva, 2009; Hansen, Noesen, Nador, & Harel, 2018; Orima & Motoyoshi, 2023). In a supplementary analysis (see Supplementary Figure S1A), we tested the corollary of this approach. We thus trained the model to detect the number and position of opened doors from the spatial memory task. When then tested on the spatial memory remaining EEG data it performed better than chance level (mean accuracy, 29.18 ± 6.19, significantly above the 12.5% chance level) (95% CI, 1.25–20.16; *p* = 0.014). However, when we tested it on the scene memory the model was no longer able to detect affordances (mean accuracy, 13.85 ± 2.40; *p* = 0.942). Finally, we compared classification accuracies of both models (i.e., the one trained and tested on spatial memory and the one trained and tested on scene memory) to see if affordances were represented more distinctly when participants had no prior knowledge of the position of the goal (Supplementary Figure S1B). The second model outperformed the first. t_(58)_ = 3.16, *p* < 0.001, 95% CI, 2.54–11.29, suggesting that affordances are represented more distinctly when participants had no prior information about the goal location. This finding aligns with the idea that participants may process affordances differently depending on task demands, emphasizing task-relevant affordance (i.e., the one hiding the goal) when required to recall goal positions.

**Figure 6.**
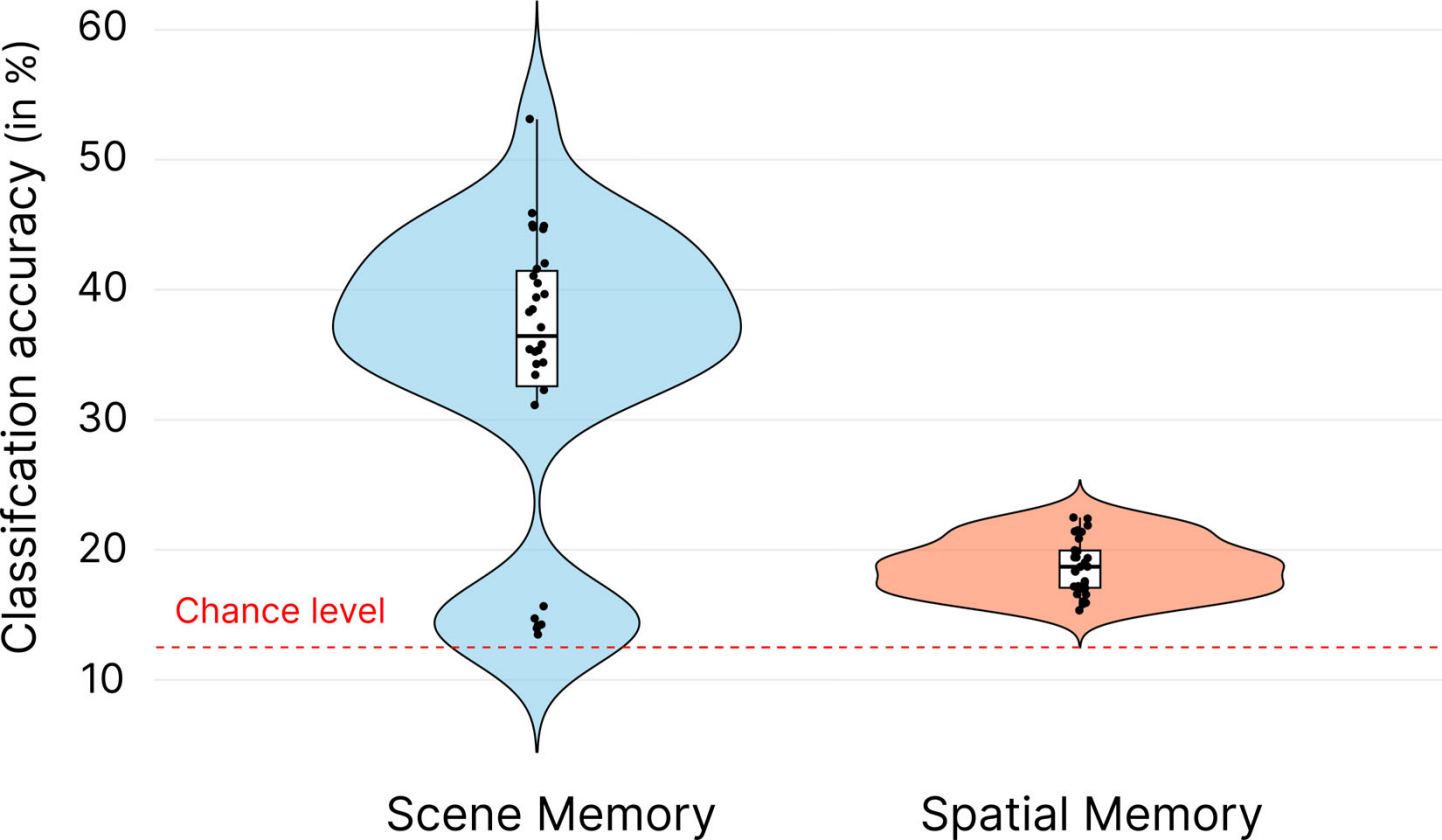
Accuracy of the CNN, trained on the data from scene memory task to detect the color of the wall (chance level at 12.5%), in the scene memory or the spatial memory task.

Finally, we aimed to test the specificity of the modulation by contextual information. We hypothesized that contextual information about goal position may selectively influence the extraction of scene features related to this information (i.e., navigational affordances), but not other unrelated features (e.g., the color of the wall). To this end, we conducted a supplementary analysis using a CNN trained to classify the color of the wall, a scene feature present in both tasks. Similar to the previous analyses, we trained a CNN on EEG data from participants performing the scene memory task, and then tested it on unseen EEG data from the same task as well as on data from the spatial memory task, to assess the generalization of the information supporting classification (Figure 6). For the scene memory task, the CNN achieved an average accuracy of 34.34 ± 11.18%, significantly above the 12.5% chance level (95% CI, 13.75–25.87; *p* < 0.0001). However, this time, when tested on EEG data from the spatial memory task, the accuracy averaged 18.75 ± 2.09%, which was still statistically above chance (95% CI, 0.31–9.65; *p* = 0.019). These results suggest that, unlike affordance detection, EEG information supporting the classification of wall color generalizes to some extent across tasks. This finding thus supports the interpretation that contextual information specifically modulates neural correlates of the extraction of scene features associated with that information. This final analysis is particularly important because, in our previous interpretation, the absence of generalization was attributed to neural activity modulation, which could have been affected by other external factors. Although the ability of the wall color model to generalize reduces these concerns, a cautious interpretation of the results is still warranted.

## Discussion

In this study, we investigated the temporal neural dynamics underlying visual perception during scene processing and its interaction with visuospatial memory information. Our results demonstrate that a CNN trained on EEG data can accurately classify several aspects of visual perception, including the color of the wall within the scene, navigational affordances, and the location of a previously learned target, primarily based on early occipitoparietal brain activity (between 100 and 230 ms). To further support the potential influence of top-down information suggested by this common time window of integration, we showed that a model trained on EEG data to decode affordance-related information in a scene memory task failed to generalize to the task in which participants had learned the position of a goal. In contrast, another model trained to detect wall color performed significantly above chance in the same context. Taken together, these results highlight the strong influence of top-down information related to prior spatial knowledge on early occipitoparietal neural activity during scene perception.

### Navigational affordances and visuospatial memory processing share a common early integration time window

The Grad-CAM analyses highlighted occipitoparietal activity at approximately 200 ms as the most important contributor to the classification of navigational affordances within the scene, consistent with previous findings by Harel et al. (2022). In their study, the authors also used a multivariate approach and suggested that the location of affordances may be represented even earlier, at approximately 130 ms. In the present study, we did not distinguish between the position and number of affordances, but found that activity at approximately both 100 ms and 200 ms contributed to the classification of affordances, with the latter time window playing a more important role. This early extraction of the position of available pathways for movement may reflect the brain's ability to rapidly construct high-level representations of visual scenes based on categorical distinctions and global spatial properties (Orima & Motoyoshi, 2021, 2023). In support of this point, studies combining magnetoencephalography measurements with deep neural network analyses have demonstrated a hierarchical progression of scene representations, integrating information from low-level features to higher-order scene features within 200 ms (Cichy, Khosla, Pantazis, & Oliva, 2017). These findings suggest that the processing of affordances from visual scenes occurs as early as other perceptual processes, bridging the gap between scene perception (e.g., encoding the number of affordances) and action (Djebbara et al., 2019; Harel et al., 2022).

Interestingly, our results suggest that higher-level information, such as spatial contextual information about the target location, is also represented in this early occipitoparietal activity. This common integration time window is consistent with several lines of evidence for top-down influences on early visual processing during scene and object perception (Enge et al., 2023; Klink, Kaiser, Stecher, Ambrus, & Kovács, 2023; Steel, Billings, Silson, & Robertson, 2021; Steel, Garcia, Goyal, Mynick, & Robertson, 2023; Steel, Silson, Garcia, & Robertson, 2024). For example, Enge et al. (2023) demonstrated that prior knowledge of an object's function shapes early cortical markers at approximately 200 ms and influences higher-order visual perception (Greene & Hansen, 2020; Groen et al., 2018). Similarly, Klink et al. (2023) reported that scene familiarity modulates early EEG activity, suggesting a role for learned scene context in shaping early visual processing. The current results support this interpretation by highlighting the critical role of visuospatial memory-derived information in shaping early visual markers of scene processing. Furthermore, these results offer novel insights into a common early time window for integrating both bottom-up and top-down information, emphasizing the dynamic interplay between scene perception and prior visuospatial knowledge. To extend these findings, future studies could examine whether similar modulation occurs when participants repeat the same task before and after learning contextual information, for example, in a scene memory task where the goal location is learned but not retrieved. However, this makes it impossible to assess whether the information was encoded successfully, because participants are not tested on this knowledge, which is why we opted for the current protocol.

### Visuospatial memory immediately influences the early neural activity of affordance extraction

To investigate the influence of top-down modulation on early neural correlates of scene perception, we examined how prior learning of the target location affects the classification abilities of the CNN in decoding affordance information. To test this, we trained a model that accurately decoded affordances in the scene memory task, confirming that the extraction of navigational affordances is represented in early occipitoparietal activity (Dwivedi et al., 2024; Harel et al., 2022). However, this model failed to detect affordances after participants learned the path to the goal in the spatial memory task, even though the presented images were exactly the same. This finding suggests that the neural correlates of navigational affordances, including their number and location, are modulated by the learning of spatial contextual information about these affordances. In a previous analysis of the same dataset, we reported at the behavioral level that increasing the number of affordances decreased participants' accuracy in the spatial memory task only (Naveilhan et al., 2024). We interpreted these results as the consequence of information related to prior spatial knowledge interacting with the automatic encoding of navigational affordances. The present neural results strengthen this interpretation, revealing a modulation of the early neural makers of affordances extractions once contextual information is learned. Critically, this effect cannot be attributed merely to task differences that would hinder the model's ability to generalize, as demonstrated by a control analysis showing significant generalization between scene memory and spatial memory tasks in a model similarly trained to decode wall colors. This lack of modulation observed for wall color features is consistent with previous findings by Hansen et al. (2018), which showed that processing of low-level visual information is rapid and minimally affected by observer-based goals. Thus, our results indicate a modulation of neural activity that is specific to scene features associated with goal location. Importantly, this effect emerges rapidly, suggesting that such modulation does not require extensive learning. This directly extends the findings of Enge et al. (2023), who proposed that semantic knowledge about objects (i.e., knowing how to use an object) influenced the early neural marker of their visual processing. Our results extend this idea to scene perception, where knowing that a goal is located behind one of the opened doors modulates early neural markers of scene processing. These findings on the interaction between scene feature integration and prior spatial information during navigational affordance processing illustrate the dynamic and complex nature of visual scene perception. They also extend the principle of top-down facilitation, originally described in object recognition. For example, Bar et al. (2006) showed that coarse visual information triggers early orbitofrontal cortex activity approximately 50 ms before recognition-related responses in high-level visual areas, thereby facilitating subsequent perceptual analysis. By analogy, knowing that a goal lies behind one of the doors likely initiates frontal feedback that could primes occipitoparietal circuits to extract the task relevant navigational affordance (see Figure 4 for this temporal pattern), highlighting the need to consider a more integrated action-oriented approach for visual perception (Nau et al., 2024).

### Perspective and limitations

These findings on the interaction between scene feature integration and prior spatial information during navigational affordance processing illustrate the dynamic and complex nature of visual scene perception. From a broader perspective, this is consistent with the predictive coding framework for efficient perception (Peelen, Berlot, & de Lange, 2024; Rao & Ballard, 1999) and the ecological basis of affordances (Gibson, 1979). This notion emphasizes the intrinsic connection between action and perception, highlighting the role of perception in guiding action, and is consistent with the notion of active inference, which views action and perception as integrated processes that work to minimize prediction error (Friston, 2010; Friston, FitzGerald, Rigoli, Schwartenbeck, & Pezzulo, 2017; Kaplan & Friston, 2018; Pezzulo & Cisek, 2016). Consistent with this theoretical framework, our results suggest that spatial memory content modulates the early neural correlates of navigational affordance processing to support efficient, goal-directed visual information processing.

Despite these interesting results it is important to acknowledge some limitations related to the methodology used here. Even if we addressed most of the limitations raised in previous similar work (i.e., using an unbiased automatic selection of hyperparameters and testing against chance level the accuracy of our models), certain methodological points merit further evaluation. In particular, the use of a fixed convolutional architecture could be enhanced by implementing nested cross-validation to optimize a deep ConvNet model, such as that proposed by Schirrmeister et al. (2017). This would allow for the adaptive tuning of temporal and spatial filter parameters, which can significantly influence the timing and spatial distribution of Grad-CAM activations. Additionally, gradient-based attribution methods often emphasize high-frequency components, potentially leading to misleading interpretations (Simonyan, Vedaldi, & Zisserman, 2014; Sundararajan, Taly, & Yan, 2017). To mitigate this, EEG analyses should maintain realistic electrode topographies rather than treating channels as independent features. Further research could also leverage integrated tools like the newly developed MEEGNet (Arthur, Annalisa, Harel, Irina, & Karim, 2025), which merges CNNs with Grad-CAM for seamless magnetoencephalography/EEG decoding, enhancing workflow efficiency and providing transparent analysis from EEG data preprocessing to interpretability.

Finally, it is important to consider alternative explanations for the modulation of affordance processing. The first concerns potential task-related difference, such as variations in task difficulty or cognitive load between the spatial and scene memory tasks. However, control analyses of theta activity over frontomedial electrodes activity, a well-established marker of cognitive load (Cavanagh & Frank, 2014) help to mitigate most concerns related to this issue (see Supplementary Figure S2). This interpretation is further supported by the fact that the task-classification model can also determine whether participants successfully learned the spatial information (see Supplementary Figure S3), suggesting that features allowing the classification are indeed related to the acquisition of this information. A second explanation arises from the findings of Castelhano and Witherspoon (2016). They demonstrated that knowledge of an object's function significantly increases search efficiency by directing attention to functionally relevant areas within a scene (Bouwkamp, de Lange, & Spaak, 2024). In our paradigm, participants may have explored the visual scene differently, and possibly directed their attention towards the task-relevant affordance (i.e., the opened door containing the goal to retrieve). Even though participants in our design had to focus on the wall to identify their color to perform the task, future studies incorporating eye tracking could help to disentangle these possibilities and clarify this potential confound.

## Conclusions

In conclusion, this work supports the notion that, during scene perception, neural processes associated with behaviorally relevant tasks, such as retrieving the position of a goal hidden behind a door, share a common time window of integration, approximately 100 to 250 ms over occipitoparietal regions, with processes representing visual information, such as navigational affordances. The present results also demonstrate an immediate influence of the newly learned spatial knowledge on the early neural activity associated with scene processing, modulating even the first stages of visual scene perception. Thus, knowing where to go may shape what you see.

## Data availability

The complete set of data, stimuli generated and code used for preprocessing, and CNN are available on the OSF repository: https://osf.io/5cqxy/?view_only=2957dac1a1304c2db6e1fb3b056c5008.

## Supplementary Material

Supplement 1
