# Supplementary material for: Where do I go? Decoding temporal neural dynamics of scene processing and visuospatial memory interactions using convolutional neural networks
Source: J Vis. 2025 Aug 28;25(10):15. doi: 10.1167/jov.25.10.15 (PMC12400970; doi:10.1167/jov.25.10.15)
Supplement: Supplement 1 [file jovi-25-10-15_s001.docx]

**Supplementary materials**

**Supplementary 2:**

**Supplementary Figure 1.** **A.** Classification performance of a model trained on the Spatial Memory task to detect affordances in visual scenes. The model performed significantly above chance when tested on the Spatial memory unseen data (in coral on the figure; mean accuracy = 29.18 ± 6.19, significantly above the 12.5% chance level (95% CI: [1.25, 20.16], p = 0.014), but when trained on the Scene Memory task, it no longer performed above chance (mean accuracy = 13.85 ± 2.40; p = 0.942). **B.** Comparison between two classification models trained to detect affordances: one trained and tested on Spatial Memory (same as in panel A), and the other trained and tested on Scene Memory (same as in Figure 4.A of the main manuscript). The second model outperformed the first (t_(58)_ = 3.16, p = 0.0003, 95% CI = [2.54, 11.29]), suggesting that affordances are represented in a more classifiable manner when participants had no prior information about the goal location. This aligns with the idea that participants may process affordances differently depending on task demands, emphasizing task-relevant affordance (i.e., the one hiding the goal) when required to recall goal positions (see **Figure 3.A** of the main manuscript)


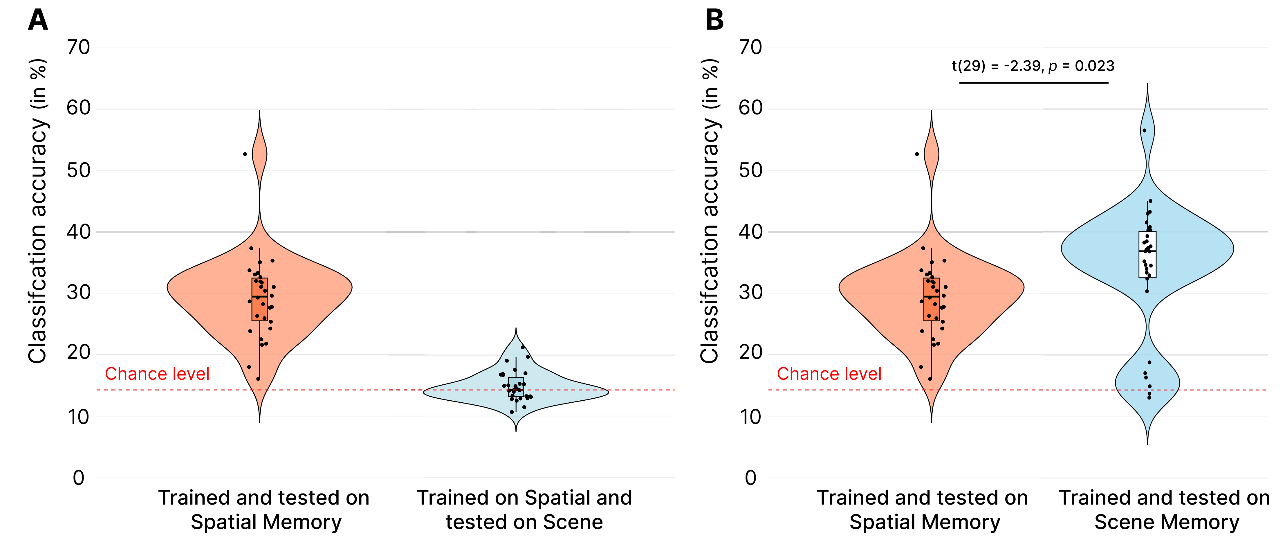


In this supplementary section, we explain why we believe that task-related differences, particularly those related to difficulty or spatial attention, are unlikely to significantly affect the classification results. One potential difference in task difficulty arises from the distinct neurocognitive processes involved: specifically, retrieving the wall color from the previous scene compared to retrieving the goal location across two environments. However, we argue that this difference is limited, based on behavioral data. Accuracy was high in both tasks (95.70% for the scene memory task vs. 93.47%, for the spatial memory task), yielding a modest difference of 2.23%. Reaction times were also comparable (535 ms vs. 551 ms, a 16 ms difference). Nonetheless, these differences were statistically significant (Accuracy: *F*_(1,125)_ = 41.82, *p* < .001, η_p_² = 0.25, 95% CI [0.13, 0.37]; Reaction Time: *F*_(1,125)_ = 25.51, *p* < .001, η_p_² = 0.15, 95% CI [0.06, 0.26]). While small, we acknowledge that these differences could reflect subtle disparities in task difficulty. To address this concern, we compared neural activity between the two conditions, focusing on fronto-medial theta (FMT), a well-established marker of cognitive load (Cavanagh & Frank, 2014). This signal could not be captured in our previous CNN-based analysis, due to the necessity of time-frequency decomposition. To overcome this, we applied superlet decomposition on a cluster of fronto-medial electrodes (FCz, Fz, Cz, FC1, FC2, F1, F2) and conducted permutation testing with FDR correction to assess potential task differences in theta-band activity. Results showed no significant differences in FMT activity between conditions (see **Supplementary Figure 2**), a result that was similar for uncorrected statistics. Taken together, these findings suggest that task-related differences are unlikely to have played a major role in driving the observed classification effects

**Supplementary Figure 2.** Control analysis of theta activity over midfrontal electrodes to investigate possible differences in cognitive load between both conditions. Permutation testing with FDR correction revealed no significant differences between conditions, supporting the interpretation that spatial attention or task difficulty may not differ substantially. Notably, similar results were observed even with uncorrected statistics.


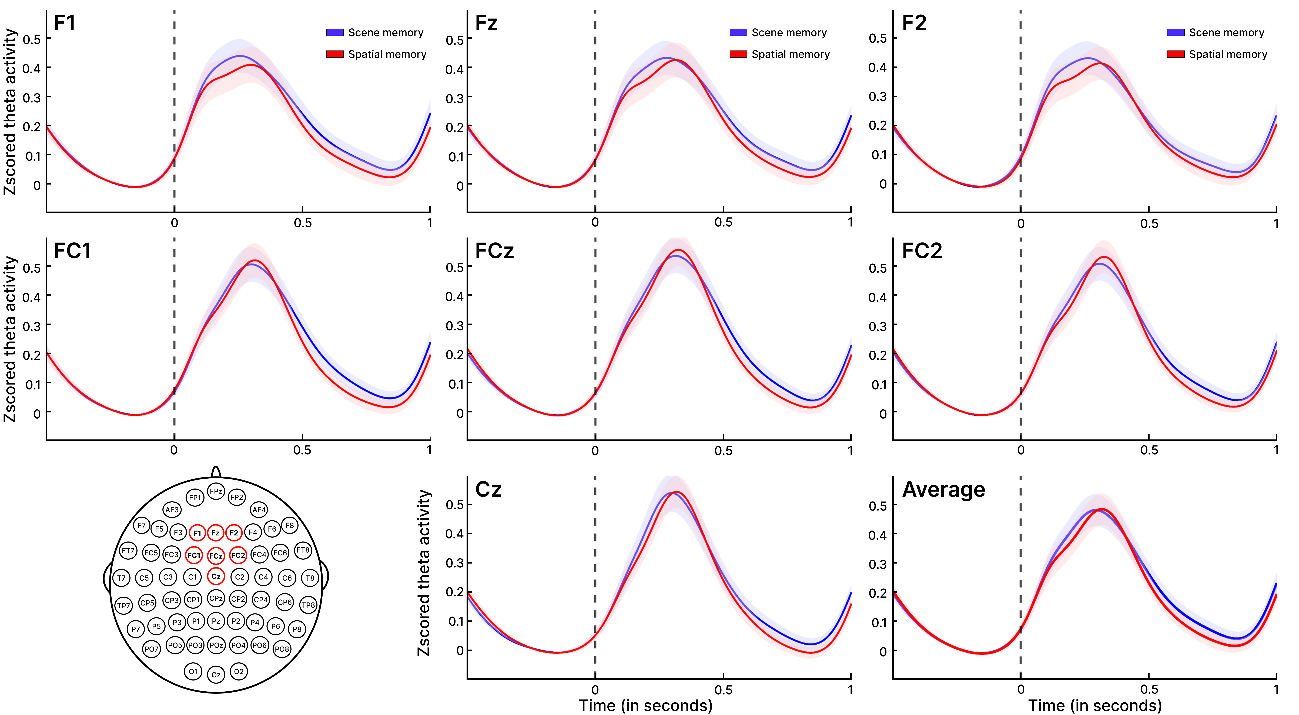

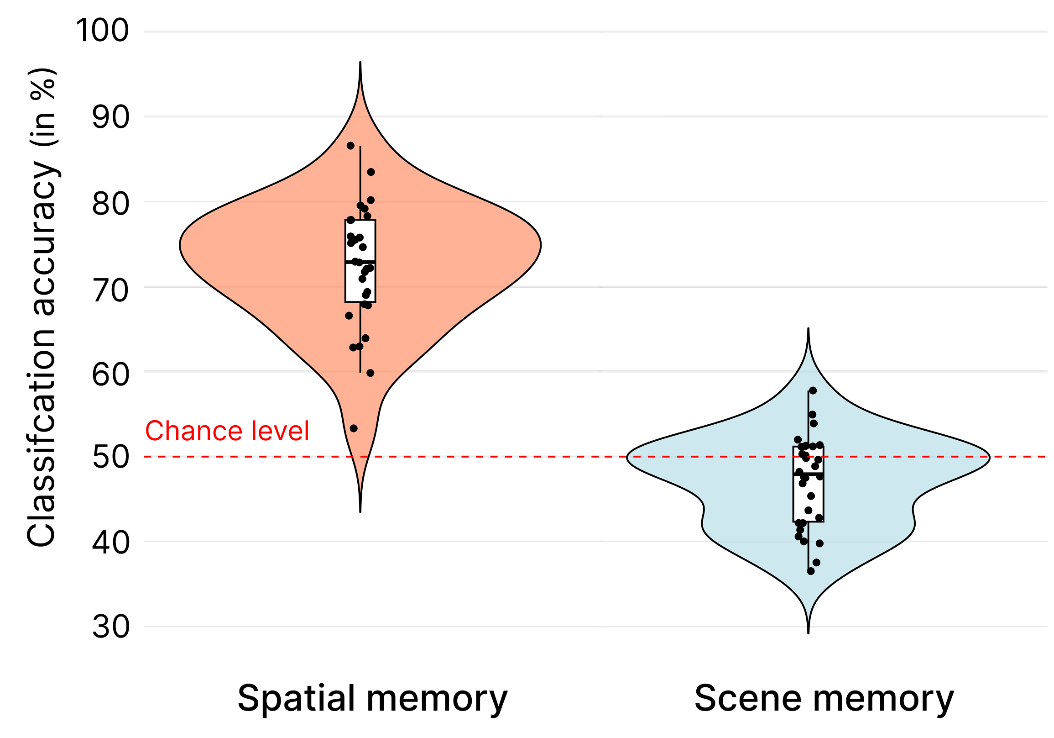


**Supplementary Figure 3.** Supplementary analysis evaluating whether the model trained to detect prior learning of contextual knowledge can also classify task performance. Here, we argue that if the model used features related to the learning of spatial knowledge, it would be able to classify when participants successfully retrieved the position of the goal (i.e., indicating that they correctly learned the position of the goal) compared to when they failed. Otherwise, if some other features would lead the classification, the model would fail to this classification. Results indicate that indeed the model trained to classify if participants learned contextual information can also classify if participants successfully retrieved or not the goal (mean accuracy = 72.52% ± 4.29; significantly above chance: 95% CI [8.54, 36.50], p < 0.001). This strongly suggests that this model indeed used features related to successful prior spatial knowledge acquisition and no other features related to potential task differences. This interpretation was finally strengthened by the fact that this model was not able to classify task performance during the scene memory task (mean accuracy = 47.12% ± 5.36; p = 0.65), in which there was no contextual information to learn.
